# The UNC-Wisconsin Rhesus Macaque Neurodevelopment Database: A Structural MRI and DTI Database of Early Postnatal Development

**DOI:** 10.3389/fnins.2017.00029

**Published:** 2017-02-02

**Authors:** Jeffrey T. Young, Yundi Shi, Marc Niethammer, Michael Grauer, Christopher L. Coe, Gabriele R. Lubach, Bradley Davis, Francois Budin, Rebecca C. Knickmeyer, Andrew L. Alexander, Martin A. Styner

**Affiliations:** ^1^Department of Psychiatry, University of North Carolina at Chapel HillChapel Hill, NC, USA; ^2^Department of Computer Science, University of North Carolina at Chapel HillChapel Hill, NC, USA; ^3^Kitware, Inc.Carrboro, NC, USA; ^4^Harlow Center for Biological Psychology, University of Wisconsin-MadisonMadison, WI, USA; ^5^Waisman Laboratory for Brain Imaging and Behavior, University of Wisconsin-MadisonMadison, WI, USA

**Keywords:** neuroimaging, non-human primate, macaque, computational atlases, magnetic resonance imaging, diffusion tensor imaging, brain development

## Abstract

Rhesus macaques are commonly used as a translational animal model in neuroimaging and neurodevelopmental research. In this report, we present longitudinal data from both structural and diffusion MRI images generated on a cohort of 34 typically developing monkeys from 2 weeks to 36 months of age. All images have been manually skull stripped and are being made freely available via an online repository for use by the research community.

## Introduction

Brain maturation is a complex process driven by cell proliferation and differentiation, myelination, and the growth of neurons and their connections during the first years of life. The initial increase in brain connections is followed by a process of dendritic pruning and loss of synaptic contacts, presumably shaping and sculpting a more efficient network of connections that are continuously remodeled throughout life (Engert and Bonhoeffer, 1999; Stepanyants et al., 2002; Lebel et al., 2008). Although neurobehavioral maturation has been studied extensively, both at a functional level (behavior) and at a structural and mediating level (cellular anatomy and physiology), information at the level of neuroanatomical connectivity and about maturational changes during the peripubertal years is not as comprehensive. This information is of special interest because it provides insight into a different aspect of neural malleability and also captures a second period of major change leading up to the adult phenotype. Furthermore, understanding developmental changes in the brain will ultimately help us to better understand the etiology of psychiatric conditions that first present clearly in adolescence, such as depression, and to devise targeted therapies for neurodegenerative brain disorders with an onset later in adulthood.

Non-human primate models are widely used to provide comparative information associated with human neuropathology (Glatzel et al., 2002; Grant and Bennett, 2003; Machado and Bachevalier, 2003; Lebherz et al., 2005; Sullivan et al., 2005; Barr and Goldman, 2006; Lubach and Coe, 2006; Segerstrom et al., 2006; Bennett, 2008; Williams et al., 2008). Advantages include the biological similarity of monkeys and humans, such as the gestation of a single offspring, a prolonged in utero development, and the maturational stage of the neonatal brain at birth. Among non-human primate models, the rhesus macaque *(Macaca mulatta*) has been the most widely used monkey to investigate the neural substrates of human behavior due to its phylogenetic closeness to humans (Lacreuse and Herndon, 2009) and the potential to examine more complex cognitive functions and social behavior associated with encephalization (Price and Coe, 2000). Furthermore, primate models allow for brain imaging at the very early, critical stages of neurodevelopment when it is challenging to recruit and image young human participants under rigorous experimental control. The use of monkeys in a laboratory setting also facilitates environmentally controlled studies with all infants raised similarly. Additionally, rhesus macaques show hemispheric asymmetry and sex differences in brain size and maturational rates during adolescence similar to humans. For more than 40 years, this species has also been used to evaluate how disturbances of the early rearing environment can induce behavioral abnormalities and influence brain development (Harlow et al., 1971).

MRI studies have significantly expanded our knowledge of human brain development during childhood and adolescence through several large-scale cross-sectional (Pfefferbaum et al., 1994; Caviness et al., 1996; Reiss et al., 1996; Courchesne et al., 2000; De Bellis et al., 2001) and longitudinal studies (Giedd et al., 1999; Sowell et al., 2004; Lenroot and Giedd, 2006). Critical periods in human brain development have been identified. In contrast, there are still some lacunae in our knowledge of non-human primate development, especially with regard to the maturational changes during the peripubertal years. While there are detailed neuroanatomical descriptions of early brain maturation in the monkey (Rakic and Goldman-Rakic, 1982), less information is available on the normal postnatal maturation of the monkey brain, with the exception of certain brain regions, such as the visual cortex. The prevailing view from studies in humans is that the total brain volume undergoes a rapid non-linear increase during childhood and reaches a maximum size around puberty. Gray matter and white matter follow distinct structure-specific developmental trajectories (Jernigan and Tallal, 1990; Schaefer et al., 1990; Reiss et al., 1996; Giedd et al., 1999; Gilmore et al., 2007a; Knickmeyer et al., 2008). While human studies suggest that postnatal cortical development is very heterochronous, postmortem studies suggest that cortical development in monkeys is more synchronous (Rakic et al., 1986; Bourgeois and Rakic, 1993).

Studies in humans indicate that there is marked sexual dimorphism in the central nervous system during childhood and adulthood. The most consistent findings include: larger volume of the cerebrum in males, higher proportion of gray matter to white matter in females, relatively greater volume of the amygdala in males, and relatively greater volume of the caudate and hippocampus in females (Dekaban, 1978; Filipek et al., 1994; Caviness et al., 1996; Giedd et al., 1996, 1997; Reiss et al., 1996; Nopoulos et al., 1997; Filipek, 1999; Gur et al., 1999; Goldstein et al., 2001; Good et al., 2001; De Bellis et al., 2001; Allen et al., 2003; Gilmore et al., 2007b; Knickmeyer et al., 2008). Studies in macaques have shown similar developmental trajectories (Knickmeyer et al., 2010; Hunsaker et al., 2014). However, it is important to note that there have been some conflicting findings in the macaque with Payne et al. finding no sex differences in the hippocampus or amygdala, although hemispheric asymmetries were present (Payne et al., 2010). Additionally, in humans, males and females also differ in specific developmental growth trajectories. Total cerebral volume, caudate volume, and gray matter volume in the frontal and parietal lobes peak earlier in girls than in boys (ages vary depending on region), a pattern which may relate to sex differences in the timing of puberty. In adolescence, white matter increases more rapidly in males than females (Lenroot et al., 2007). These sex differences in neurobiology may be relevant to certain neurodevelopmental pathologies, which frequently indicate that there are marked sex differences in risk, phenotypic expression, and treatment response (Szatmari et al., 1989; Moffitt, 1990; Gur et al., 1996; Häfner et al., 1998; Chakrabarti and Fombonne, 2001; Moffitt and Caspi, 2001; Baird et al., 2006; Kulkarni et al., 2008). Research on monkeys can be particularly informative about the role of pubertal onset in determining these brain changes because females reach puberty1–3 years before males. In addition, adult rhesus monkeys also display large sex differences in brain size (Falk et al., 1999). This dimorphism occurs in part because the male brain continues to grow significantly after puberty (Franklin et al., 2000). However, some areas, such as the amygdala, continue to be of similar size in male and female monkeys.

**Figure 1:**
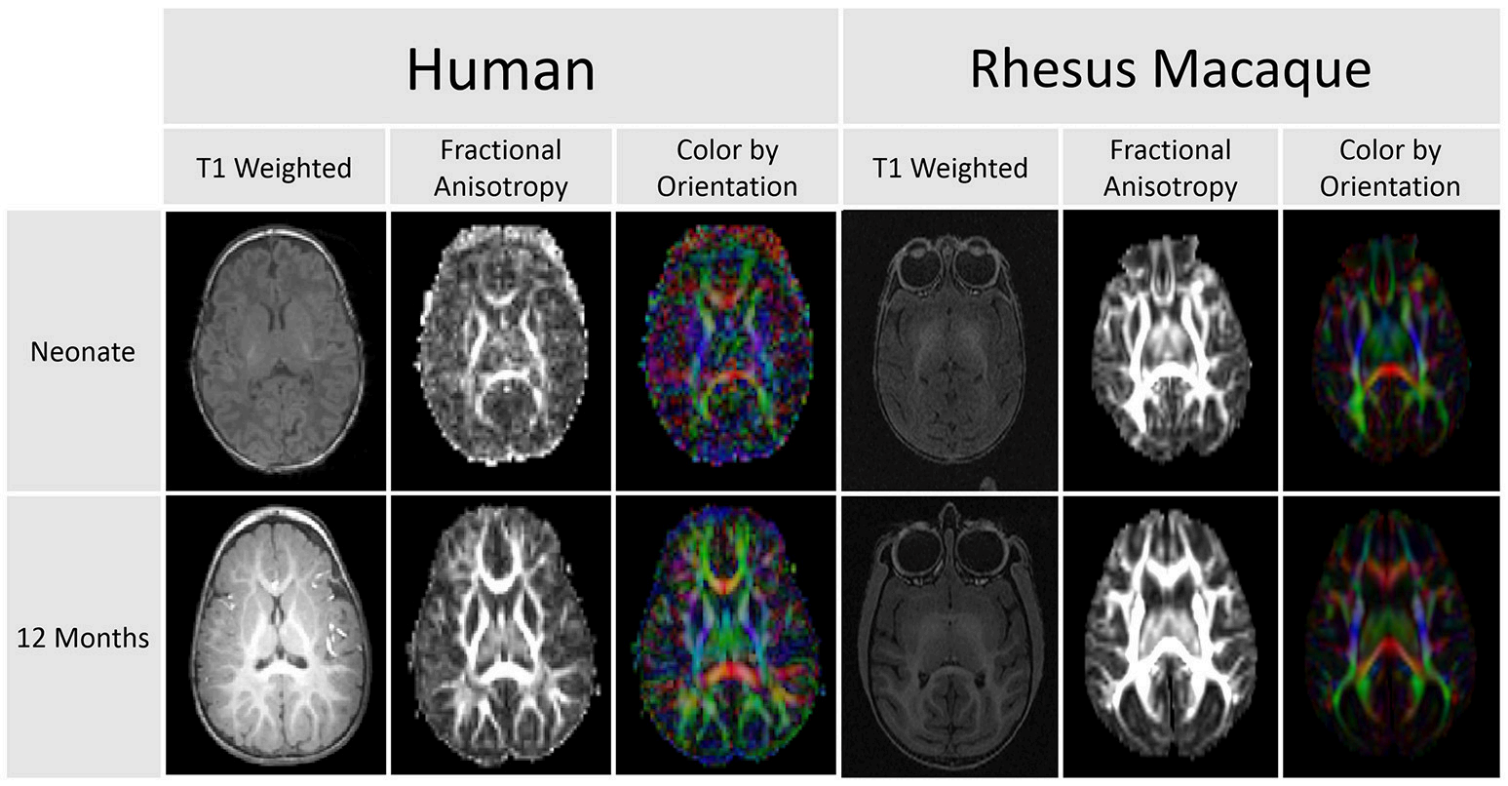
**This comparison of DTI scans for humans and rhesus macaques demonstrates that diffusion anisotropy is observable before a clear distinction between the white and gray matter is visible in the T1 weighted image**. This difference in clarity and delineation is apparent in both the human and macaque images.

As portrayed in Figure 1 below, white matter changes are observed throughout the first two decades of life and beyond in humans (Lebel et al., 2008) and can be measured by DTI. Few developmental studies using DTI have been published for non-human primates. Makris et al. (2007) investigated changes in white matter fiber bundles with aging, but this study was done in elderly macaques. They reported reductions in fractional anisotropy (FA) in cortico-cortical association fibers with age and general agreement with observations in humans. Styner et al. (2008) explored changes in the developing macaque brain using an atlas-driven brain parcellation and showed increases in FA, particularly in the corpus callosum, as well as a decrease in mean diffusivity. Combining structural MRI and DTI is a particularly useful approach to investigate brain development over the full course of neurodevelopment. While DTI provides sufficient white matter contrast at the earliest stages of brain development, it is more difficult to analyze the fetal and young infant brain with structural MRI due to weak intensity contrast and contrast inversion.

Scott et al. also investigated typical brain development in the rhesus macaque (Scott et al., 2016). However, DTI data were not presented and those data have not been made publically available at this time. A DTI atlas characterizing typical brain development in the rhesus macaque was made publically available, however, it had a relatively low resolution and few gradient directions (Zakszewski et al., 2014). Additionally, this study only made available the final atlas and not any of the individual scans.

The current paper marks the major release of new data to our publicly available MRI database characterizing macaque brain development. It is hoped that this freely available resource will be of great utility to others in the neuroimaging field and enable more effective use of monkeys as translational models in both basic neuroscience and clinical research. Non-human primates, such as the rhesus macaque, provide a unique opportunity to study normal brain maturation and behavioral development in a comparative manner, especially in early infancy, where crucial information is still missing. Additionally, this database will continue to be updated as we process the data, create atlases, and perform fiber tracking on the DTI data.

## Methods

### Subjects

The subjects for these analyses were 34 rhesus monkeys (*Macaca mulatta*) reared and housed at the Harlow Primate Laboratory (HPL) at the University of Wisconsin-Madison. The HPL maintains a 500+ monkey colony dedicated to producing infants, with a capacity for generating approximately 70–100 infants per year. Complete rearing and health histories, as well as pedigrees, are known for all animals. Adults were mated with single sires to ensure the required number of subjects was met. Only healthy infants and juveniles were assigned to this project.

The housing consisted of stainless steel caging (each 0.9 × 0.9 × 0.9 m), where each female monkey lived with her infant, either individually or as a pair with another adult female in double cages. The housing arrangement is conducive to rigorous experimental control over pregnancy conditions, food consumption, and other environmental variables. Animals were fed a standardized diet of commercial biscuits and fruit supplements and foraging devices for enrichment. Water was available *ad libitum*, the temperature was controlled at 21.5°C, and the light/dark cycle was maintained at 14:10 with lights on at 06:00.

All infants were reared normally by their mothers until weaning occurred at 6–7 months of age. Afterwards, the older juveniles were housed in small social groups or as a pair to provide companionship. This rearing and housing strategy was designed to facilitate their normal socialization and to ensure standardized rearing conditions.

The research protocol was approved by the Institutional Animal Care and Use Committee (IACUC). Care and treatment of the animals at HPL are designed to meet and exceed the guidelines promulgated by the National Institutes of Health Guide for the Care and Use of Laboratory Animals. The quality of the research findings is predicated on the high quality of care.

### Subject pedigree

Of the 34 macaques included in this study, three of the subjects were maternal half siblings, while eighteen were paternal half siblings (same father). Six of the eighteen paternal half siblings had two paternally related half siblings. No subject had more than two half siblings or both a paternal and maternal half sibling. Additionally, none of the subjects were full siblings. See Table 1 for full pedigree information.

**Table 1:** **Subject Demographics**.

**Subject**	**Gender**	**Birth weight (grams)**	**Matriline**	**Patriline^†^**	**Age of first scan (Days)**
001	Female	438	1	1	360
002	Male	590	2	2	363
004	Male	590	4	4	482
005	Female	452	5	4/5	601
006	Male	648	6	6	498
007	Male	495	7	7	261
008	Female	460	8	8	197
010	Male	626	10	9/10	197
011	Female	425	11	4	498
012	Female	580	12	12	195
013	Male	616	13	13	148
014	Female	463	14	14	146
015	Female	420	15	9/14	132
016	Male	672	16	1	118
017	Male	460	17	3/17	95
018	Female	609	18	44	103
019	Female	496	19	1	121
020	Male	480	20	20	64
021	Female	500	21	21	74
022	Male	570	22	4/9	57
023	Female	488	23	23	54
024	Female	510	24	24	26
025	Male	540	25	23	27
026	Female	728^*^	26	26	87
027	Male	541	1	27	97
028	Male	582	28	9	19
029	Male	546	29	24	122
030	Female	516	30	2	116
031	Male	500	31	12	32
032	Female	492	32	32	17
033	Female	568	33	9	28
034	Male	476	18	27	16
035	Male	622	35	35	13
036	Male	565	36	10	18

* *Subject was weighed 1 month after birth*.

† *In some cases the exact sire is not known*.

### Inclusion criteria for dams

The breeding females chosen for this study were adults between 5 and 15 years of age and multiparous. The females were healthy, did not have a prior history of a Caesarean section, and had successfully reared at least one infant.

### Gestational and infant growth parameters

Female macaques were mated with a designated sire to ensure knowledge of paternity. Maternal and infant weight data were obtained at periodic intervals to document normal growth. All monkeys scanned were healthy, and any illness would have been evaluated by the attending veterinarian.

### Scanning schedule

Thirty-six subjects were initially assigned to this study which involved a series of up to five scans at scheduled intervals. Two subjects were ultimately excluded during the course of the study: one because of an adverse reaction to the anesthetic used for scanning, and a second because of illness during early postnatal development prior to first scan. All subjects retained in the final analyses were scanned five times, except for one subject scanned only four times due to a scheduling issue. There were additionally two subjects scanned only once and then subsequently dropped from the study due to adverse reactions to the anesthetic. However, these single scans were included in this analysis because they provided information on the critical early infancy period (<3 months of age).

**Figure 2:**
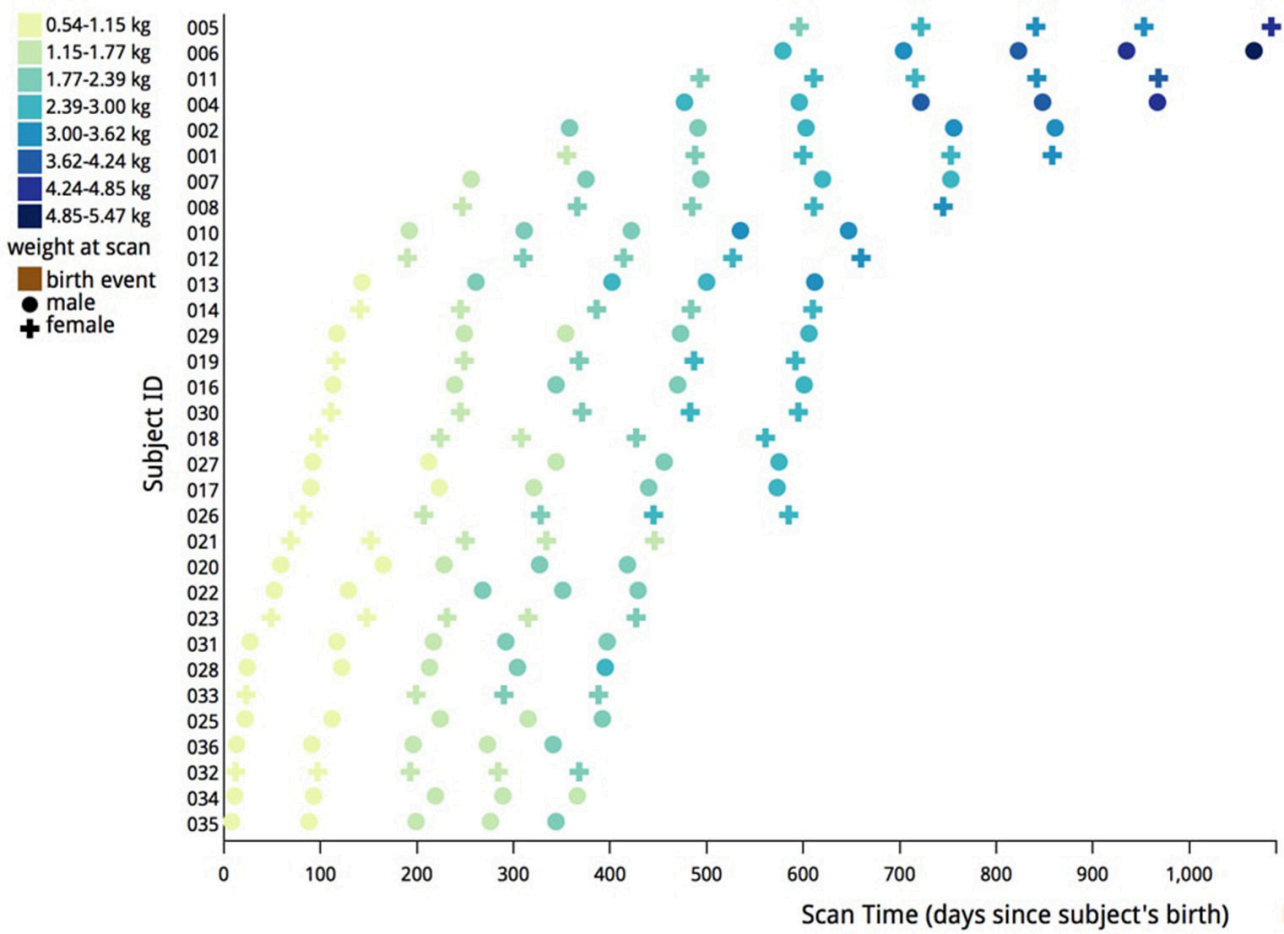
**Illustration of actual scanning schedule used in this analysis**. Each data point represents a scan of either a male (circle) or female (cross) subject. The data points are color coded based on the weight in kilograms of each subject at the time of the scan. Most subjects were scanned at five different times to create the data for this study.

The original scanning schedule was as follows: for subjects with their first scan when younger than 4 months, a 3 month scan interval was used. For subjects with their first scan at 4 months or older, a 4 month scan interval was used. Two animals were scanned at each time point, one male and one female. Four animal pairs were scanned beginning at 2 weeks of age, two pairs beginning at 1 and 2 months of age, and one pair scanned beginning at 3, 4, 5, 6, 8, 12, 16, and 20 months of age. During the course of this project, a small adjustment was made in the schedule (see Figure 2). The scanning schedule was amended such that two pairs of subjects were first scanned when younger than 5 months in order to increase the sample size for early infancy.

This staggered scheme also allowed for testing any possible influence of the sedation or handling procedures. Another advantage of this experimental design is the relatively large sample size, with the repeated scanning enabling us to conduct both longitudinal and cross-sectional analyses.

### MRI acquisition

Scans were performed on a GE MR750 3.0T scanner (General Electric Medical, Milwaukee WI) using the human 8-channel brain array coil at the Waisman Laboratory for Brain Imaging and Behavior at the University of Wisconsin-Madison. In order to ensure a safe plane of anesthesia and recovery, the scanning protocol for animals younger than 6 months lasted approximately 30 min. For subjects older than 6 months of age, the scanning procedure was extended to slightly <1 h to improve the signal-to-noise ratio on the diffusion weighted image (DWI) scan.

### Anesthesia

Subjects were given a pre-anesthetic (ketamine hydrochloride 10 mg/kg I.M.) for transport to the MRI facility. For infants younger than 6 months of age, immobilization during the scan was achieved with inhalant isoflurane. Older subjects were immobilized throughout the scanning procedure by an initial administration of ketamine hydrochloride (10 mg/kg I.M.) followed by dexdomitor (0.01 mg/kg I.M.). The effects were reversed at the end of the session by administering atipamezole (0.15 mg/kg I.V.). The plane of anesthesia was monitored with a pulse oximeter to track heart rate and oxygen saturation in both younger and older subjects.

### Structural imaging

High-resolution 3D T1-weighted imaging was performed using an axial Inversion Recovery (IR) prepared fast gradient echo (fGRE) sequence (GE BRAVO) (*TI* = 450 ms, *TR* = 8.684 ms, *TE* = 3.652 ms, FOV = 140 × 140 mm, flip angle = 12°, matrix = 256 × 256, thickness = 0.8 mm, gap = −0.4 mm, 80 percent field-of-view in phase encoding direction, bandwidth = 31.25 kHz, 2 averages, total time = 10:46 min) provided an effective voxel resolution of 0.55 × 0.55 × 0.8 mm across the entire cranium. The T2-weighted scan was performed using a sagittal 3D CUBE FSE sequence (*TR* = 2500 ms, *TE* = 87 ms, FOV = 154 × 154 mm, flip angle = 90°, matrix = 256 × 256, 90 percent field of view in the phase encoding direction, slice thickness = 0.6 mm, gap = 0 mm, bandwidth = 62.5 kHz, ARC parallel imaging with a factor of 2 acceleration in both phase encoding and slice encoding directions, total time = 6:36 min) across the cranium was acquired with a voxel resolution of 0.6 × 0.6 × 0.6 mm.

### Diffusion imaging

The following DWI protocol was used in order to obtain the highest possible resolution for the DTI: *TR* = 8000 ms, *TE* = 65.7 ms, FOV = 16.7 mm, matrix = 128 × 128, 2.6 mm slice thickness with 1.3 mm slice overlap (resolution 1.3 × 1.3 × 2.6 mm^3^), upsampled to an voxel dimension on the scanner of 0.65 × 0.65 × 1.3 mm^3^, ASSET parallel imaging with an acceleration factor of 2, and a coronal slice orientation. The overlapping slices provide finer spatial sampling than the original resolution (similar to the Fourier interpolation in-plane), and also provide higher SNR. There were 120 unique gradient directions acquired with *b* = 1000 s/mm^2^ and ten baseline images with *b* = 0 s/mm^2^. A scan time of approximately 17.5 min was required to obtain these images.

### Data transfer

All MRI data were transferred electronically via an existing web-based upload center from the Waisman Imaging Center to the UNC NIRAL, where they were integrated into study data and automatically backed up on local servers.

### Image analysis

**Figure 3:**
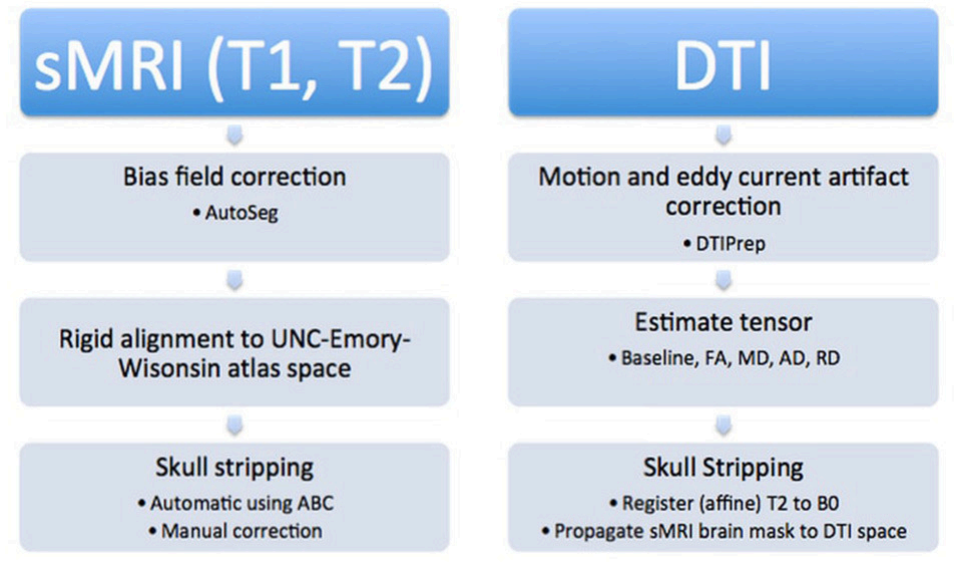
**Structural images were processed first by performing bias field correction, followed by rigid alignment to the atlas**. Additionally, automatic and manual skull stripping was done. The DTI images were corrected for motion and eddy current artifacts, tensors were calculated, and finally, the T2 weighted image was registered to the baseline image and the brain mask was applied to the diffusion data to complete skull stripping.

All of the images were segmented automatically using an existing image analysis pipeline. However, many of the automatically generated segmentations were inaccurate and all segmentations were examined manually and corrected. Subjects younger than 6 months of age required the most significant manual corrections. Manual correction of the segmentations was performed using the ITK-SNAP^1^ tool. An overview of the pipeline is described below and can be seen visually in Figure 3.

#### Structural MRI pipeline

AutoSeg^2^ was used to perform the processing of the structural images. First, a bias field correction for the T1 and T2 images, respectively, was applied using the N4 method (Tustison et al., 2010). T1 images were aligned to an external T1 atlas (Emory-UNC atlas at 12 months) with rigid body registration (normalized mutual information based registration with BRAINSfit^3^). Then, the T2 images were aligned to their atlas-registered T1 images. As part of this realignment, the T1 and T2 images were re-sampled in atlas space to 0.2375 × 0.2375 × 0.2375 mm resolution. Atlas Based Classification^4^ (ABC) was applied to the registered images for expectation maximization based tissue classification (white matter, gray matter, cerebrospinal fluid, background) (Van Leemput et al., 1999). Tissue segmentation results were used to generate binary brain masks. Finally, all automatically generated brain masks were corrected manually by a human expert (JY).

#### Diffusion imaging pipeline

The DWI arrived at UNC NIRAL in the DICOM file format. These files were converted to the NRRD format using a tool known as DWIConverter^5^ that is a built-in module of 3D Slicer.^6^ 3D Slicer is an open source software package for image visualization and analysis. The converter tool within 3D Slicer computes DWI based on the b-matrix information in the DICOM header.

Diffusion MRI quality control and motion and distortion correction was performed using our quality control (QC) tool, DTIPrep,^7^ that is also available as a built-in module of 3D Slicer (Liu et al., 2010; Oguz et al., 2014). This tool allows for image viewing and cropping, checking *b*-values and gradient vectors, noise filtering, evaluating correlation intensities to check for brightness and venetian blind artifacts, detection of vibration artifacts, and motion and eddy current artifact correction (Farzinfar et al., 2013). Motion and eddy current correction is done first for baseline (*b* = 0) images via a rigid transform based iterative average image computation over all baseline images, followed by affine registration of the diffusion weighted images to the final baseline average image. This QC step is done automatically using a protocol file containing all parameter settings for DTIPrep (Oguz et al., 2014; Verde et al., 2014). The images were examined visually after the automatic QC to exclude additional gradient directions that contained motion or artifacts. The average remaining gradient directions was 116 ± 11. There were no associations in gradient direction exclusion with respect to age or gender. Three subjects had partial acquisitions but were still included in the database for the interested parties. Once the visual QC was completed the DTI images were automatically generated from the corrected DWI data via weighted least square estimation.

3D Slicer was used to perform a visual QC on the corrected DTI images. This was done to verify the correct directional encoding, ensure an adequate signal-to-noise ratio (SNR), and perform fiducial tractography to verify the presence of all major tracts as described in Verde et al. (2014).

Skull stripping was performed by applying the brain mask created in the structural processing pipeline to the diffusion data. For each subject, its T2 weighted image was registered to the corresponding baseline image via standard normalized mutual information based affine registration, and the previously manually edited structural brain mask was propagated with this transformation to generate the DTI brain mask. This DTI brain mask was employed during the computation of the masked diffusion tensor data using weighted least squares fitting (3D Slicer external DTIProcess module).^8^ Eigenvalue maps (λ_1_, λ_2_, λ_3_) as well as diffusion property maps of fractional anisotropy ($FA=\sqrt{\frac{1}{2}}\sqrt{\frac{{\left(\lambda_{1}\;-\;\lambda_{2}\right)}^{2}\;+\;{\left(\lambda_{2}\;-\;\lambda_{3}\right)}^{2}\;+\;{\left(\lambda_{3}\;-\;\lambda_{1}\right)}^{2}}{\sqrt{\lambda_{1}^{2}\;+\;\lambda_{2}^{2}\;+\;\lambda_{3}^{2}}}}$), mean diffusivity ($MD=\frac{1}{3}\sum_{i}\lambda_{i}$), axial diffusivity (*AD* = λ_1_) and radial diffusivity ($RD=\frac{\lambda_{2}\;+\;\lambda_{3}}{2}$) were computed from the masked DTI data.

## Results

**Figure 4:**
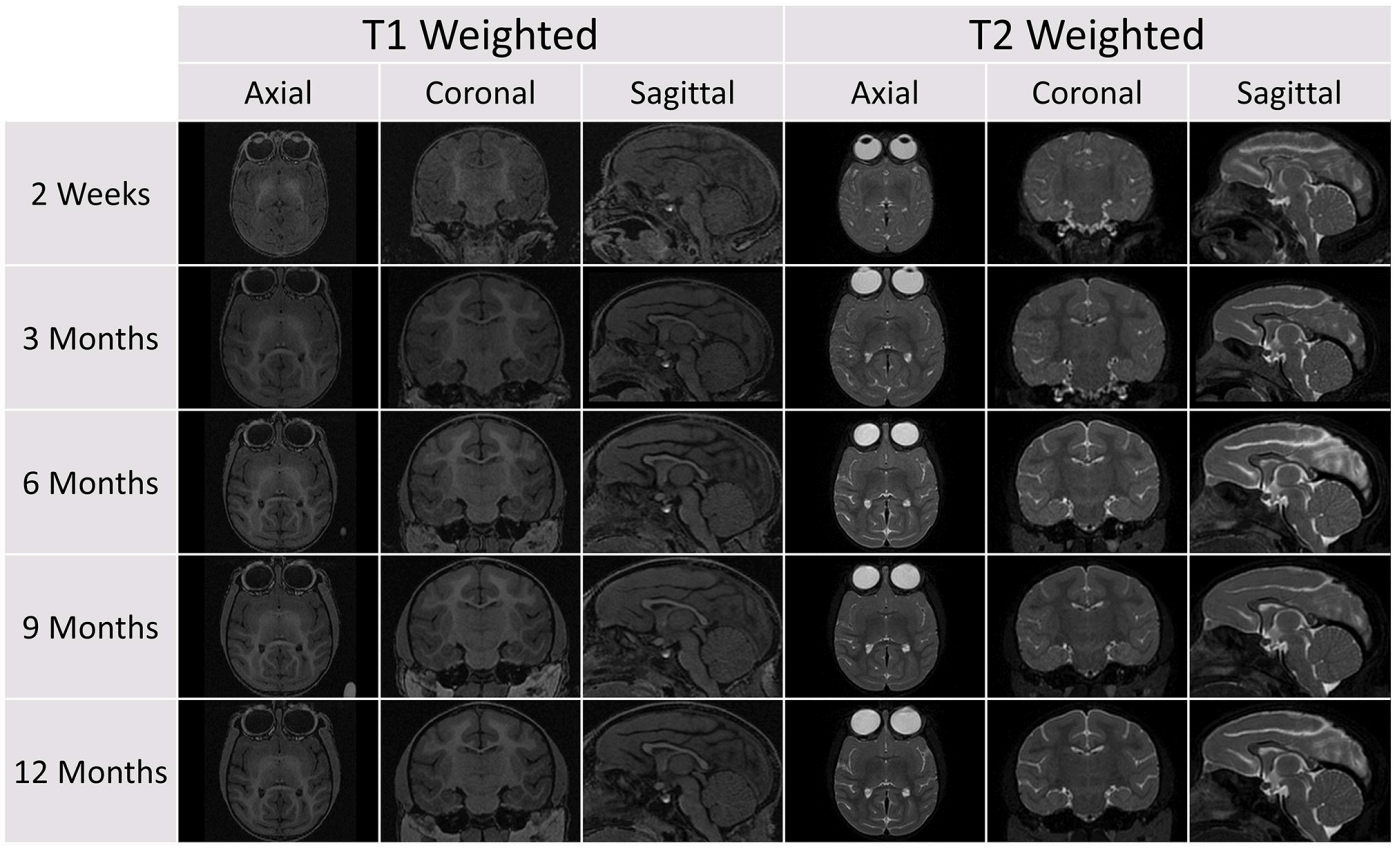
**A representative sample of the serial images acquired for a single subject in the database (Subject 032)**. Each subject was scanned at five age points. At each time point, a T1 and T2 scan was obtained and the image above shows axial, coronal, and sagittal views for each scan sequence.

Using the methods outlined above, we were able to successfully obtain and process the serial images of brain development in rhesus monkeys. Both the structural and DTI data have been made freely available via the Girder repository.^9^ A representative example of the processed structural MRI data is shown below in Figure 4. All of the raw, unprocessed, structural and DWI data are also now available in this repository. The data can be downloaded in whole or in part using a standard secure FTP client, such as Transmit, Cyberduck, Filezilla, or the Unix or Mac command line utility.

**Figure 5:**
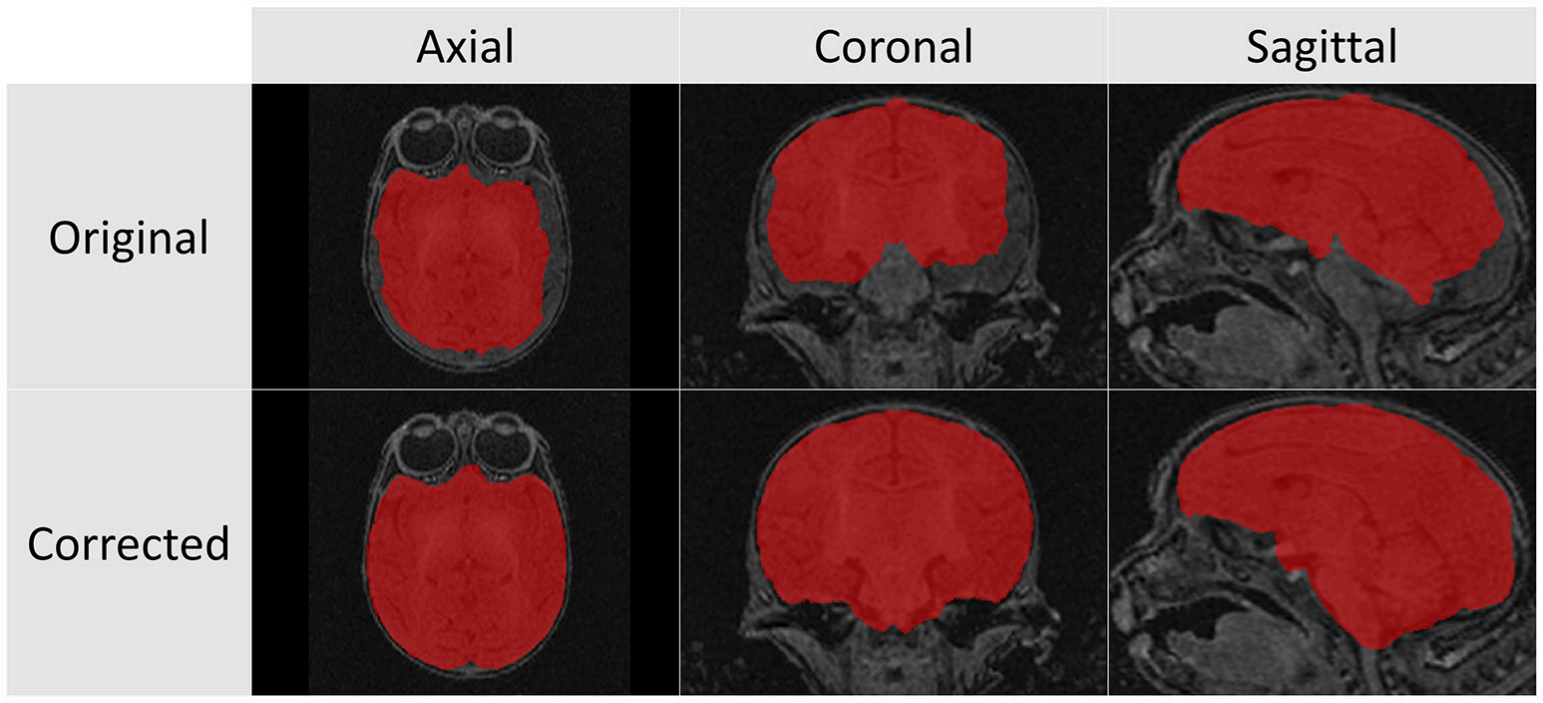
**These T1 images illustrate the difference between the automatic segmentation (top)** and the manual corrected results **(bottom)** when performed on scans of a 2 week infant monkey. Automatic segmentations on younger subjects required significantly more manual corrections in part due to poor tissue contrast.

The structural scans required significant manual corrections after the automatic segmentation was applied. These manual corrections were performed by an expert rater (JY). A representative case of these manual corrections can be seen in Figure 5. Due to the rapid growth of the brain during the early stages of development there is a high degree of variation with regard to brain structure between subjects. Because of this population variance, automatic segmentation is less effective in younger subjects and all images of subjects younger than 6 months required significant manual corrections. For the subjects older than 6 months only minimal manual corrections were needed.

**Figure 6:**
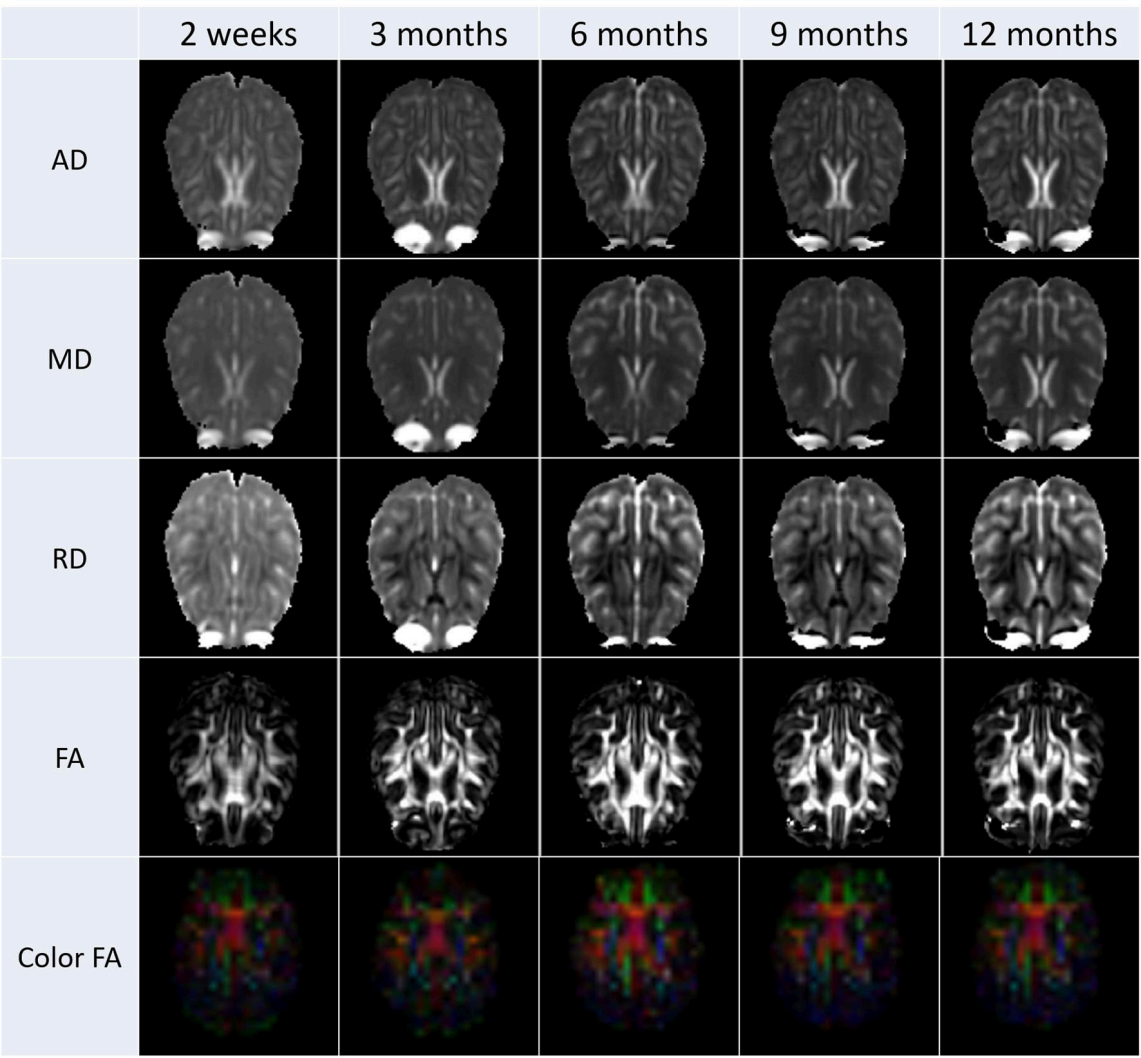
**These images are an illustrative example the DTI results calculated from scans from all subjects**. Property maps were generated at each scanning time point.

After the diffusion scans were processed, diffusion property maps were generated for each subject. These maps included AD, RD, FA, and color by orientation. Figure 6 illustrates the property maps that were computed.

## Discussion

**Figure 7:**
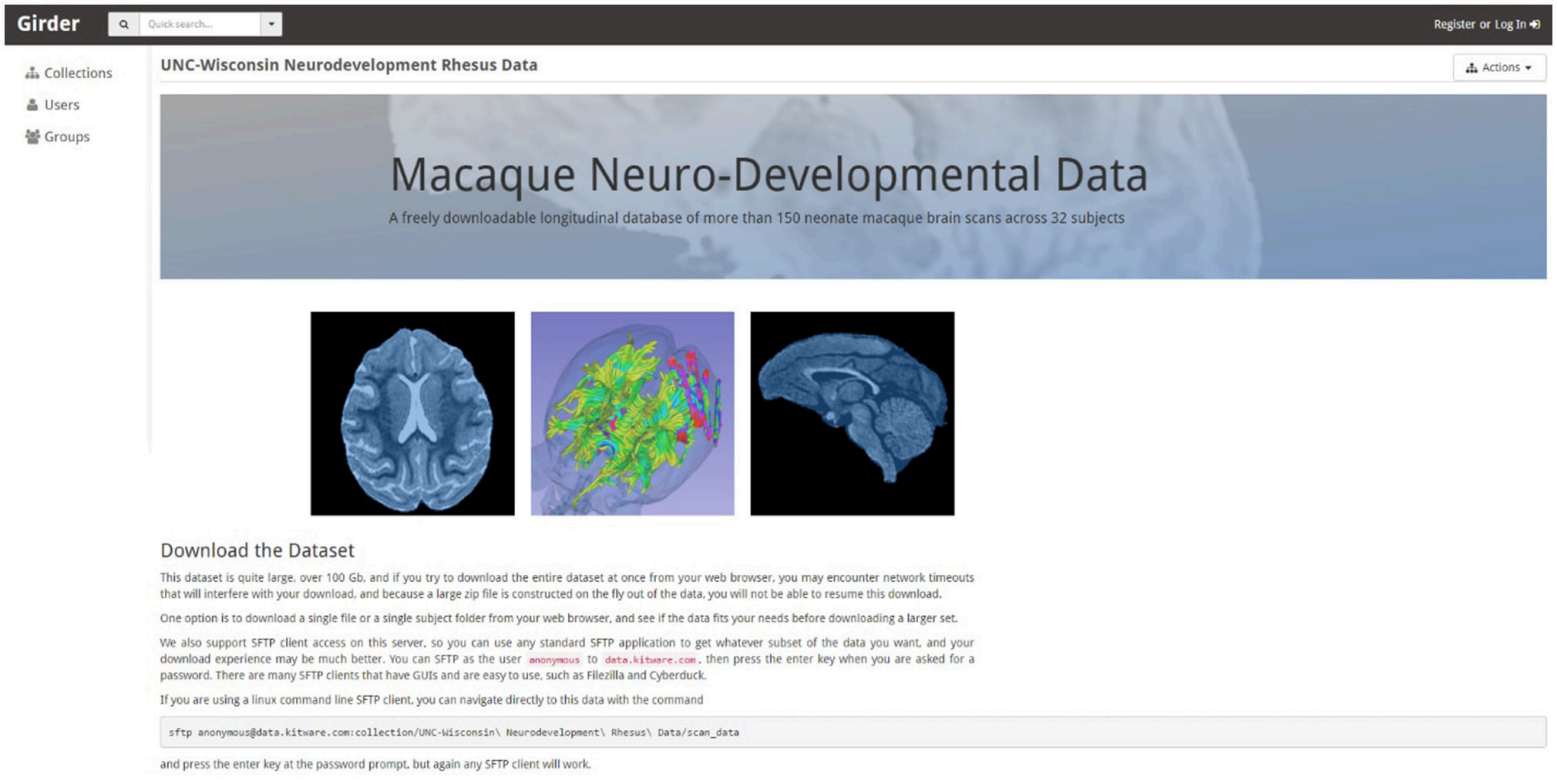
**All data from this study is being made publically available and can be accessed from the homepage shown above**. The data can be downloaded in whole or in part using a standard SFTP client. This repository will continue to be updated as we process the data.

This paper coincides with the public release of the processed data online. The data are available on Girder, an online data repository developed by Kitware (Figure 7). Prior to this release, the raw, preprocessed data were already available on Girder. These raw data have been downloaded 285 times since they were made available in April 2015. Further refinements to the UNC-Wisconsin Neurodevelopment Rhesus Database are still ongoing. In the future, age-specific brain atlases will be constructed at each time point using other derived data obtained from this study. These atlases will also be publicly disseminated for use by others. We also plan to perform full brain tractography and will release that data after validation and post-processing of the tracts.

This normative database provides a significant resource for the neuroimaging field and creates a foundation for investigations of abnormal development that may occur in the context of drug or teratogen exposure or following atypical rearing or nutrition conditions. The scan data from this project span two transitional periods likely to be critical in the etiology of mental illnesses. First, there is the period of rapid synaptic elaboration during infancy, the non-human primate equivalent of early childhood when neurodevelopmental conditions, such as autism spectrum disorder first present. Second, the scans of older juveniles and sub-adult monkeys in the 2–3 year old age range were obtained during the dynamic peripubertal period, when dendritic pruning occurs. Our data clearly demonstrate the emergence of significant sex differences in brain volumes and rate of maturation at this time. In humans, this stage is when gender differences in depression and eating disorders emerge, as well as when other psychopathologies like schizophrenia become more manifest. Our database provides both longitudinal and cross-sectional reference points against which to calibrate abnormal maturation during these vital points in neural development. It would be challenging to schedule an equivalent series of scans for children and adolescents, as well as to control all aspects of their diet and living conditions that can influence the maturing brain. These studies in humans are only now beginning, such as the baby connectome project which has just received funding. In addition, we were able to ensure the absence of prenatal and delivery complications, and exclude any monkey with significant clinical illness during the course of the study. We anticipate that this database will contribute to novel discoveries and look forward to seeing these data being productively incorporated into other analyses in the future.

## Author contributions

JY, YS performed the analysis and interpretation of the data. MN, RK contributed to the conception of the study. MG, FB, and BD contributed to the analysis of the data. CC, GL, and AA contributed to the conception of the study and the acquisition of the data. MS contributed to the conception of the study, analysis, and interpretation of the data. All authors contributed to the drafting and revising of the manuscript, as well as giving final approval for publication.

### Conflict of interest statement

The authors declare that the research was conducted in the absence of any commercial or financial relationships that could be construed as a potential conflict of interest.

## Footnotes

^1^http://www.itksnap.org/

^2^https://www.nitrc.org/projects/autoseg/

^3^https://www.nitrc.org/projects/multimodereg/

^4^https://www.nitrc.org/projects/abc

^5^http://wiki.slicer.org/slicerWiki/index.php/Documentation/4.4/Modules/DWIConverter

^6^http://www.slicer.org

^7^http://www.nitrc.org/projects/dtiprep

^8^http://www.nitrc.org/projects/dtiprocess

^9^https://www.nitrc.org/projects/uncuw_macdevmri
